# Impact of horizontal and vertical posterior pole steepness on the dimensions of idiopathic macular hole

**DOI:** 10.1186/s40942-025-00700-6

**Published:** 2025-06-23

**Authors:** Niroj Kumar Sahoo, Jay Chhablani, Ashika Patil, Ninan Jacob, Mudit Tyagi, Srinivas Rao Podili, Rahman Khan Pathan

**Affiliations:** 1 Anant Bajaj retina institute, L V Prasad Eye Institute, Kode Venkatadri Chowdary Campus, 521134 Vijayawada, India; 2 UPMC Eye Centre, University of Pittsburgh, Pittsburgh, PA 15213 USA; 3 Anant Bajaj retina Institute, , L V Prasad Eye Institute, Kallam Anji Reddy Campus , Hyderabad, 500034 India

**Keywords:** MH, Macular hole, Steepness, Curvature

## Abstract

**Background:**

Macular holes (MH) vary immensely with respect to size and shape among different individuals. In an attempt to see if the curvature of the retinal pigment epithelium has any role, we aimed to analyse the influence of horizontal and vertical posterior pole steepness on idiopathic macular hole dimensions.

**Methods:**

This was a retrospective observational study done in eyes with a diagnosis of idiopathic macular hole. Various MH parameters like baseline hole parameters like minimum linear diameter (MLD), baseline hole diameter (BHD), and hole angles were calculated. Steepness in the form of tangent angles was measured in both horizontal and vertical meridian at 6 mm and under the MH margins at 1.5 mm.

**Results:**

52 eyes of 52 patients were included. Horizontal MLD showed weak correlation with average horizontal hole angle (*r*=-0.28, *p* = 0.04); average vertical hole angle (*r*=-0.28, *p* = 0.04). Horizontal BHD showed significant correlation with the horizontal hole margin tangent apical angle (*r*=-0.46, *p* = 0.001); average horizontal hole angle (*r*=-0.45, *p* = 0.001). Vertical MLD showed correlation with average horizontal hole angle (*r*=-0.28, *p* = 0.04) and average vertical hole angle (*r*=-0.33, *p* = 0.03). Vertical BHD showed correlation with horizontal hole margin tangent apical angle (*r*=-0.38, *p* = 0.004) and vertical hole margin tangent apical angle (*r*=-0.31, *p* = 0.03); vertical 6 mm tangent apical angle (*r*=-0.36, *p* = 0.01). On multivariate regression analysis, factors favouring a type 2 closure include, lower superior hole angle and higher inferior to superior tangent base angle ratio.

**Conclusion:**

Hole margin tangent angles were seen to correlate with the size and shape of the hole. Future studies with bigger sample size are required to validate it further.

## Background

Macular holes (MH) are full thickness defects in the neurosensory retina at the foveal center and is an important cause of visual loss. Idiopathic MH represents the most common subtype, typically affecting the elderly population. It is thought to be a result of abnormal traction forces by the posterior vitreous cortex at the center of the fovea. Although recent modifications in the surgical procedure have led to closure rates nearing 100%, pre-operative visual acuity remains the most important prognostic factor in these cases [1–3]. 

Several measurements and indices have been developed, that take into consideration different characteristics of the hole, to aid in predicting closure rates. Some of them include the minimum linear diameter (MLD), hole height (HH), basal hole diameter (BHD), macular hole index, hole forming factor, tractional hole index, diameter hole index, etc [4, 5]. However, in clinics, all of these parameters are used on a single horizontal scan. Since MH is a 3-dimensional structure, a single scan doesn’t provide information about all the edges of a macular hole. In a previous report, we showed the utility of enface optical coherence tomography (OCT) in analyzing the closure patterns post-operatively [6]. We found that eyes with an oval orientation, tend to close in a linear pattern more often. Also, in eyes with MLD more than 650 microns, a linear pattern of closure had a better final visual acuity than round closure. This led us to introspect, why some holes were round, while others were oval, considering the fact that all idiopathic holes arise as a result of a common pathologic process. This information could help us to predict which holes would close in a linear pattern and thus have a better outcome. We realized that the only inconsistent structural parameter which had a large inter-person variability and that could possibly influence the MH directly, was the shape of the eyeball, specifically the posterior pole curvature. The posterior pole curvature has been described before to correlate well with various structural changes in high myopic eyes and in macular holes [7–11]. With the help of our study, we aim to utilize the same principle to analyze the influence of this tangent angle (which indirectly measures the curvature or steepness) on the MH margin and its possible effect on the shape of the hole.

## Methods

This was a retrospective, multicentric (India and USA) observational study of patients with a diagnosis of idiopathic full-thickness macular holes January 2022 to January 2023. Ethical approval from the local institutional review boards were obtained [LEC-BHR-R-09-22-939 (India), and STUDY20120113 (USA)] and the study adhered to the tenets of declaration of Helsinki. Informed consent was obtained from all subjects. Eyes with epiretinal membrane (ERM) around the hole, vitreous attachment at the hole edges, operculated macular holes [only stage 3 or stage 4 macular holes (Gass classification) were included] [12], previous history of vitreoretinal surgery and eyes with poor documentation were excluded. Eyes with axial length more than 24 mm, were also excluded. Parameters such as basic demographic details and duration of symptoms, were also recorded.

### OCT analysis

**Fig. 1:**
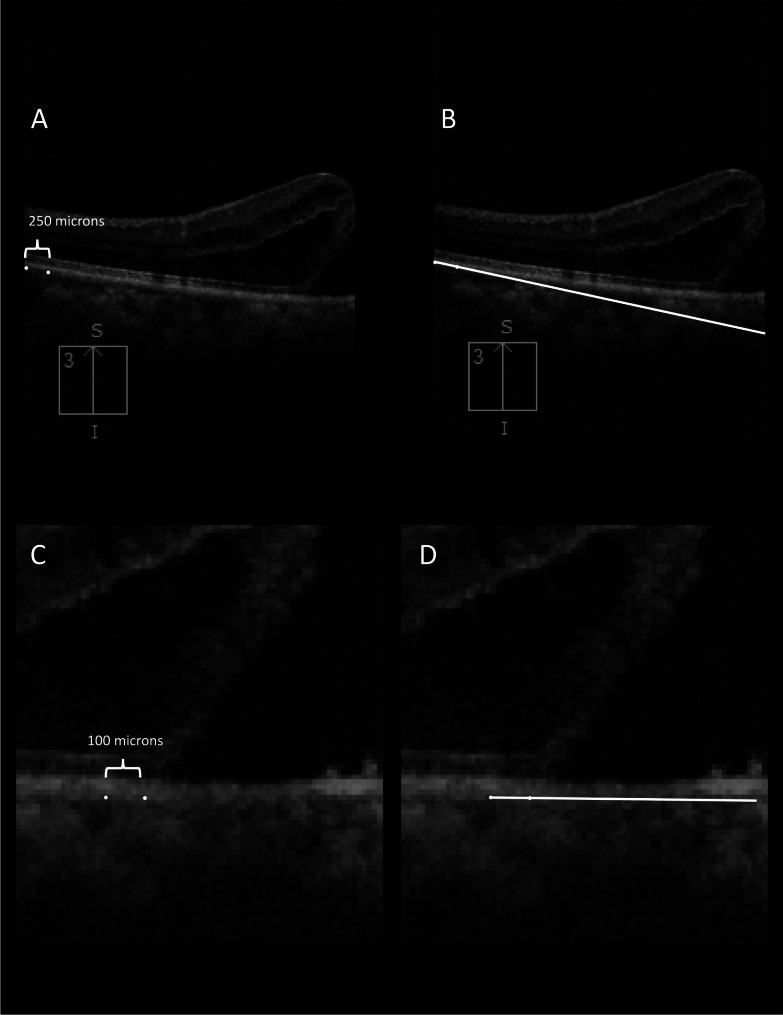
Figure showing example of how tangent was drawn. (**A**) Two points (250 microns apart) were marked at the outer border of retinal pigment epithelium (RPE) at 6 mm point. (**B**) The line joining the 2 points was taken as the presumed 6 mm tangent. (**C**) Two points (100 microns apart) were marked at the outer border of RPE below the hole margin, and (**B**) line joining these 2 points was taken as the presumed hole margin tangent

**Fig. 2:**
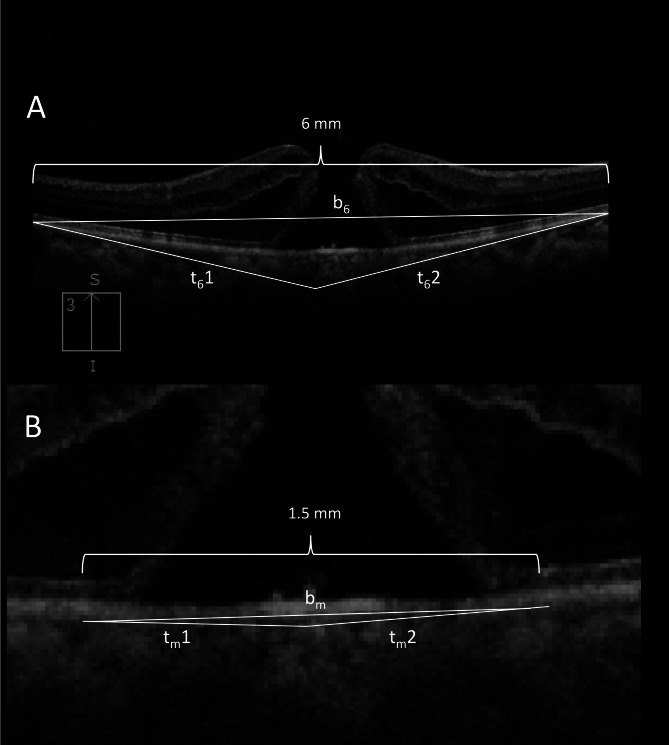
Figure showing the process of drawing the two triangles for analysis. (**A**) The two tangents at 6 mm form 2 sides of the triangle (t_6_1 and t_6_2). The base (b_6_) is formed by a line joining the 6 mm points at the outer border of RPE. (**B**) A second triangle was drawn with the two tangents (t_m_1 and t_m_2) drawn below the hole margin as the two sides, and the base (b_m_) being formed by a line joining two points below the hole margin

**Fig. 3:**
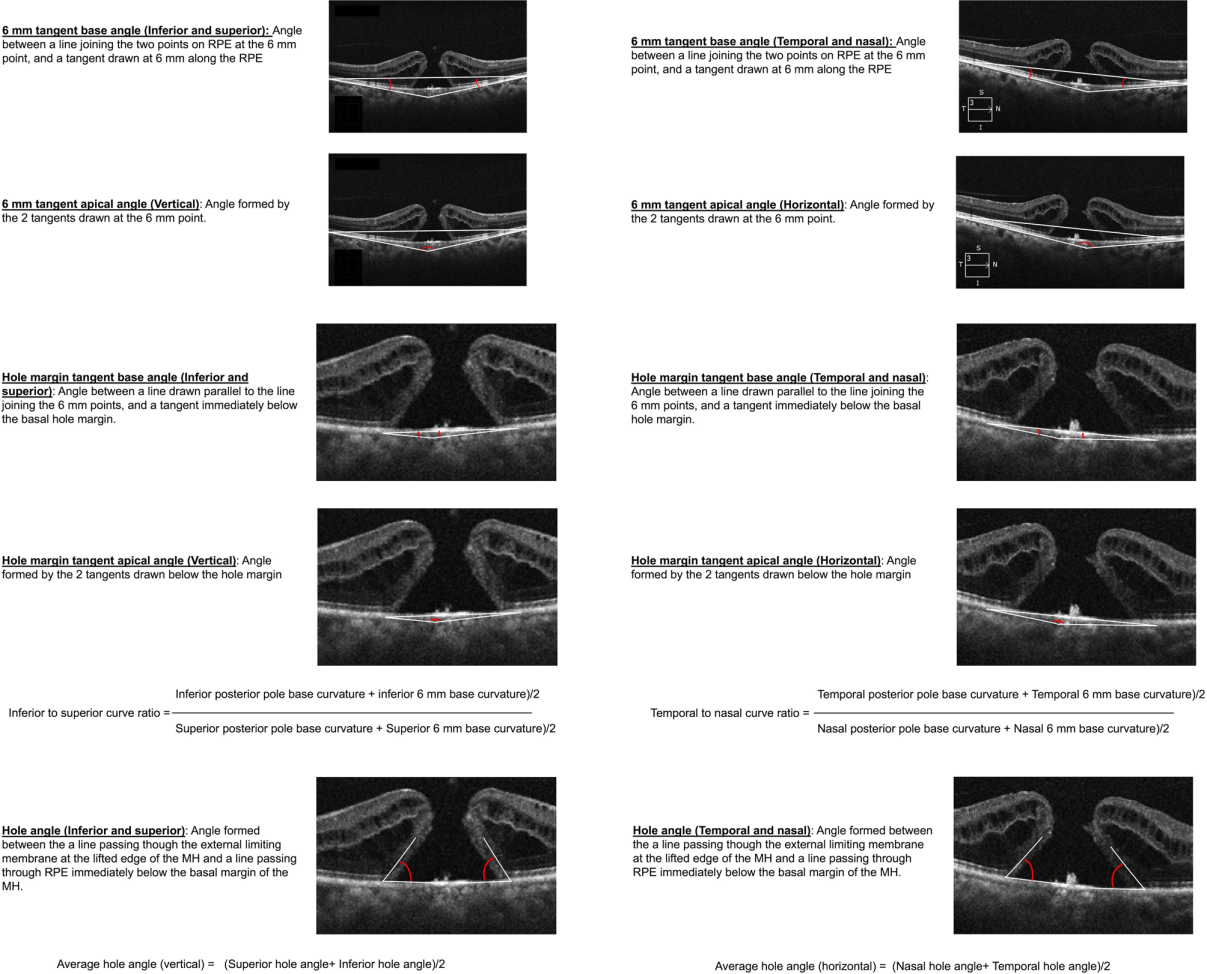
Figure demonstrating the different measurements used

**Fig. 4:**
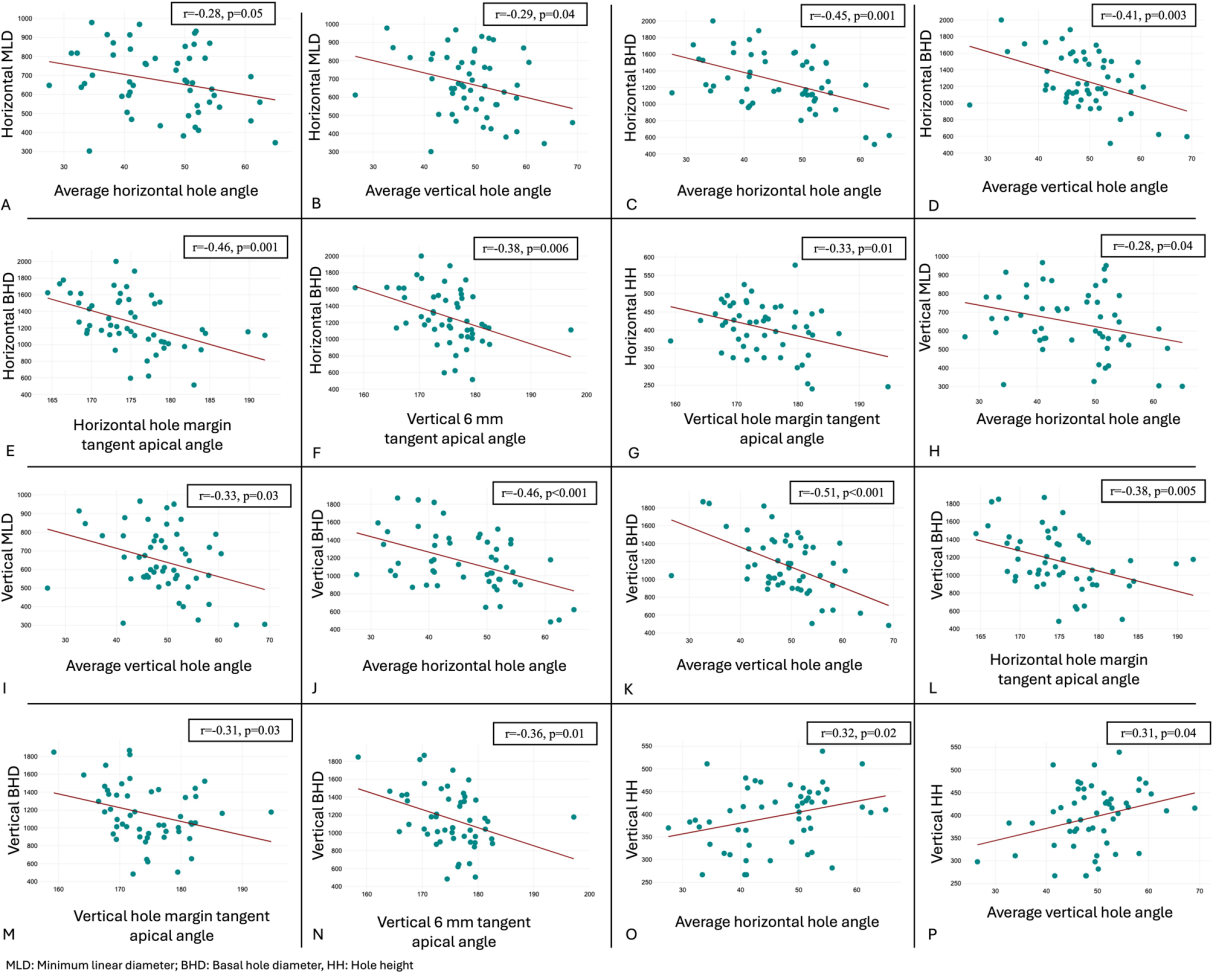
Figure showing various significant correlations for horizontal minimum linear diameter (**A**, **B**), horizontal basal hole diameter (**C**, **D**, **E**, **F**), horizontal hole height (**G**), vertical minimum linear diameter (H, I), vertical basal hole diameter (**J**, **K**, **L**, **M**, **N**), vertical hole height (**O**, **P**)

Pre-operative and postoperative OCT horizontal and vertical scans were obtained using the same OCT machine [Cirrus 6000 (Carl Zeiss Meditec, Dublin, CA)] and same protocol (6 mm HD cross), without flattening, to maintain uniformity in aspect ratio. It was made sure that the macular holes occupied the center of the scan. Hole measurements were taken in the axes with largest hole dimensions, in both the horizontal and vertical scans, and included the horizontal and vertical minimum linear diameter (MLD), hole height (HH), basal hole diameter (BHD), and hole angle (HA). Basal hole diameter was defined as the diameter of the hole at the retinal pigment epithelium (RPE) level, while minimum linear diameter was measured between the nearest points of the walls of the hole [13]. All diameter measurements were done using built-in calipers in the OCT devices. Angle measurements were done using public domain software ImageJ by Fiji (Figs. 1, 2, 3 and 4). HA was measured as the angle formed between a line passing though the external limiting membrane at the lifted edge of the MH and a line passing through RPE immediately below the basal margin of the MH (Fig. 3) [14]. Average horizontal and vertical hole angles were measured as a mean of nasal and temporal hole angles, and mean of superior and inferior hole angles, respectively (Fig. 3). The posterior pole tangent angles in the horizontal and vertical meridian, both at 6 mm point and below the hole margin, were measured. On a 6 mm horizontal or vertical OCT scan, a tangent (presumed tangent) was drawn at 6 mm, such that it passed through 2 points on the outer border of RPE, one point being at 6 mm and the other point placed ~ 250 microns posterior to the first point (this was done for nasal, temporal, superior and inferior point at 6 mm) (Fig. 1A and B). An imaginary triangle was drawn with the base as a line joining two points at the outer border of RPE at 6 mm on either side of the scan, and the tangents drawn at 6 mm (Fig. 2A). The **6 mm tangent base angle** was **(nasal**,** temporal**,** superior and inferior)** was defined as the angle between the base and the tangent at 6 mm (Fig. 3). The **6 mm tangent apical angle (horizontal and vertical)** was defined as the angle formed by the 2 tangents drawn at the 6 mm point (Fig. 3). Similarly, another tangent (presumed tangent) was drawn below the hole margins by joining 2 points, one exactly below the hole margin at around 1.5 mm (750 microns on either side of center), and another point placed ~ 100 microns anterior to it (Fig. 1C and D). A second imaginary small triangle was drawn below the hole, with the base as a line joining 2 points at the outer border of RPE at 750 microns on either side of center, and the tangents drawn (Fig. 2B). The **hole margin tangent base angle (nasal**,** temporal**,** superior and inferior)** was defined as the angle between the base and tangent below the hole margin (Fig. 3). The **hole margin tangent apical angle (horizontal and vertical)** was measured as the angle formed by the 2 tangents drawn below the hole margins (Fig. 3). The 6 mm tangent base angles and hole margin tangent base angles at each quadrant were used to get an average tangent base angle in nasal, temporal, superior and inferior quadrant (Fig. 3). Nasal to temporal tangent base angle ratio, and inferior to superior tangent base angle ratio were defined as the ratio of average nasal tangent base angle and average temporal tangent base angle, and ratio of average inferior tangent base angle and average superior tangent base angle respectively (Fig. 3). For each of the values, 2 graders (AP and NJ) measured the angles, and an average of the angles was taken into the final analysis. The eyes that underwent pars plana vitrectomy with internal limiting membrane (ILM) peeling, with or without ILM graft (free/inverted), and the type of macular hole closure after surgery were also recorded. Type 1 closure on OCT was defined as macular hole closure without any defect of the neurosensory retina, whereas type 2 closure was defined as closure of the hole with a central defect in neurosensory retina.

### Statistical analysis

Considering a correlation co-efficient value of 0.4 between ocular shape index and horizontal hole diameter from a previous study study, keeping the power at 80% and alpha error at 0.05, a sample size of 47 was reached [11]. After adding an attrition rate of 10%, a final sample size of 52 was included. Statistical analysis was done SPSS statistical software version 20 (SPSS, Inc., Chicago, IL, USA). Continuous variables were reported as mean ± standard deviation (SD). Best corrected visual acuity was converted and represented as logarithm of minimum angle of resolution (logMAR). Normality was confirmed for all variables using Shapiro-Wilk test. Between-group correlations were performed using Pearson correlation. Multivariable linear regression analysis was also carried out to assess the factors affecting the hole measurements. Interobserver and intra-observer agreement (done in a sample of 20 eyes) for the angle measurements was expressed in the form of interclass correlation co-efficient. Logistic regression analysis was performed to assess the factors affecting the type of closure (type 1 or type 2) of macular hole. All demographic parameters, hole and angle measurements were used as independent variables. A *p*-value of < 0.05 was taken as statistically significant.

## Results

A total of 52 eyes of 52 patients (17 males 35 females), with a mean age of 66.2 ± 8 years, were included. The mean axial length of the eyes was 23.08 ± 0.78 mm. The median duration of symptoms before presentation was 2.5 months (IQR 1 to 12 months).

### Macular hole and tangent characteristics (Table 1)

**Table 1:** Demographic parameters and ocular metrics

Demographic data	
**Parameters**	**Value**
Age, years*	66.2±8
Gender (Females), %	35, 67.3
Duration of symptoms, months (Median)	2.5
**Ocular metrics**	
Baseline BCVA, logMAR*	0.9±0.2
Phakic,%	27,51.9
Horizontal MLD, microns*	672.6±172.5
Horizontal BHD, microns*	1271.1±326.9
Horizontal hole height, microns*	405.8±71.6
Horizontal 6 mm tangent angle, degrees*	165.5 ± 8.5
Horizontal hole margin tangent apical angle, degrees*	175.1 ± 5.7
Average horizontal hole angle, degrees*	46.2 ± 8.7
Vertical MLD, microns*	645.1±171.5
Vertical BHD, microns*	1157.2±325.8
Vertical hole height, microns*	395.6±67.1
Vertical 6 mm tangent apical angles, degrees	175.1 ± 5.9
Vertical hole margin tangent apical angle, degrees*	174.5 ± 6.4
Average vertical hole angle, degrees*	49.1 ± 7.5

BCVA: Best corrected visual acuity; logMAR: logarithm of minimum angle of resolution; MLD: Minimum linear diameter; BHD: Basal hole diameter

*Expressed in mean ± standard deviation

The mean horizontal MLD was 672.7 ± 172.5 and the mean horizontal BHD was 1271.1 ± 326.9 microns. Similarly the mean vertical MLD was 645.1 ± 171.5 and the vertical BHD was 1157.2 ± 325.8 microns. The mean hole height were 405.8 ± 71.5 microns and 395.6 ± 67.1 microns in horizontal and vertical meridian respectively. The average values of 6 mm tangent apical angles in the horizontal and vertical OCT scans were 165.5 ± 8.5 degrees and 175.1 ± 5.9 degrees, respectively. The difference was statistically significant (*p* < 0.001). The average value to horizontal hole margin tangent apical angle was 175.1 ± 5.7 degrees and the vertical hole margin tangent apical angle was 174.5 ± 6.4 degrees. The difference was not statistically significant (*p* = 0.62).

### Correlations

**Horizontal MLD** (Fig. 4-A, B) showed weak negative correlation with average horizontal hole angle and average vertical hole angle. Multivariable linear regression did not show any significance for both the variables.

**Horizontal BHD** (Fig. 4-C, D,E, F) showed significant negative correlations with the average horizontal hole angle, average vertical hole angle, horizontal hole margin tangent apical angle, and vertical 6 mm tangent apical angle. On multivariable linear regression, horizontal hole margin tangent apical angle (regression co-efficient=-23.2, *p* = 0.04), and average horizontal hole angle (regression co-efficient=-11.2, *p* = 0.05), were the only parameters found to be significant.

**Horizontal hole height (**Fig. 4-G) showed significant negative correlation with vertical hole margin tangent apical angle.

**Vertical MLD** (Fig. 4-H, I) showed significant negative correlations with average horizontal hole angle, and average vertical hole angle. Multivariable linear regression did not show any significance for both the variables.

**Vertical BHD** (Fig. 4-J, K,L, M,N) showed significant negative correlations with average horizontal hole angle, average vertical hole angle, horizontal hole margin tangent apical angle, vertical hole margin tangent apical angle, and vertical 6 mm tangent apical angle. Average vertical hole angle (regression co-efficient=-16.7, *p* = 0.01) was the only factor found to be significant on multivariable linear regression analysis.

**Vertical hole height** (Fig. 4-O, P) showed positive correlations with average horizontal hole angle, average vertical hole angle. Multivariable linear regression did not show any significance for both the variables.

The **ratio of horizontal and vertical hole margin tangent apical angles** also showed significant positive correlation with ratio of MLD (*r* = 0.55, *p* < 0.001).

Horizontal and vertical MLD and BHD values showed good correlations with each other [horizontal MLD versus horizontal BHD, *r* = 0.7, *p* < 0.001; horizontal MLD versus vertical MLD, *r* = 0.9, *p* < 0.001; horizontal MLD versus vertical BHD, *r* = 0.71, *p* < 0.001; horizontal BHD versus vertical BHD, *r* = 0.95, *p* < 0.001; vertical MLD versus horizontal BHD, *r* = 0.74, *p* < 0.001.

There was no correlation of any of the measurements with other parameters like age, gender, duration of symptoms, baseline BCVA, and lens status.

Interobserver ICC analysis showed good agreement in terms of both hole angle measurements (co-efficient = 0.97, *p* < 0.001), and tangent angle values (co-efficient = 0.98, *p* < 0.001). Similarly, the intra-observer ICC values were 0.99 (*p* < 0.001) and 0.98 (*p* < 0.001), respectively for both measurements.

### Association with type of closure

A total of 36 eyes underwent surgery and had documented post-operative documentation. The mean age of this subset was 64.7 ± 7.5 years, and the mean pre-operative horizontal MLD was 664.3 ± 182.8 microns. The mean duration of symptoms was 5.9 ± 6.7 months and the mean baseline best corrected visual acuity (BCVA) was 0.92 ± 0.27 logMAR (Snellen equivalent of 20/160). A total of 11 eyes underwent conventional ILM peeling and 25 underwent an inverted ILM peeling. OCT and post-operative visual acuity were taken after a mean duration of 1.3 ± 0.9 months of surgery. Twenty six eyes (72.2%) had a type 1 closure while 10 (27.8%) other had a type 2 closure. Visual acuity after surgery was 0.73 ± 0.4 logMAR (Snellen equivalent of 20/125). On multivariate regression analysis (Table 2), factors favouring a type 2 closure include, lower superior hole angle and higher inferior to superior tangent base angle ratio.

**Table 2:** Factors predicting type 1 or type 2 closure

	Univariate				Multivariate			
	p-value	Exp(B)	95% C.I.for Exp(B)		p-value	Exp(B	95% C.I.for EXP(B)	
			Lower	Upper			Lower	Upper
Superior hole angle	0.01	0.85	0.77	0.98	0.01	0.76	0.56	0.95
Inferior to superior curve ratio	0.04	14.10	1.13	176.18	0.02	2478.76	3.27	1878718.61

## Discussion

In our study, we found a good correlation between various aspects of the posterior pole tangent angles with different MH measurements, both in the horizontal and vertical scan. While we did not find any direct correlation between the absolute values of MLD and the posterior pole tangent angle, we found a good correlation with the macular hole angles. On the other hand, absolute BHD and height measurements showed good correlation with the posterior pole tangent angles. We also found that the ratio of horizontal and vertical tangent apical angles, correlated well with the ratio of horizontal and vertical MLD. On analysing the closure pattern, a lower superior hole angle and a higher inferior to superior tangent base angle ratio favoured a type 2 closure.

Posterior pole curvature is a less investigated entity. Minami et al. showed that the chorio-scleral interface curvature, choroidal thickness and the axial length can affect the RPE curvature at the posterior pole [8]. Later, Müller et al. described various types of curvatures, including the dome shaped macula in a cohort of in 65,440 individuals [9]. Posterior pole curvatures have also been studied in pathological conditions like high myopia. The degree of posterior pole curvature has been shown to be directly associated with the degree of myopic maculopathy [10], and with the progression of myopia [15]. Baba et al. also demonstrated the effect of scleral imbrication on posterior staphyloma [7]. The authors found a flattening of the staphyloma curve along with reduction of axial length and improvement in macular schisis after the procedure. These studies imply a direct impact of the shape of the posterior pole on chorioretinal disorders involving the macula, although its impact on the morphology of idiopathic macular hole in non-myopic eyes has been less studied.

**Fig. 5:**
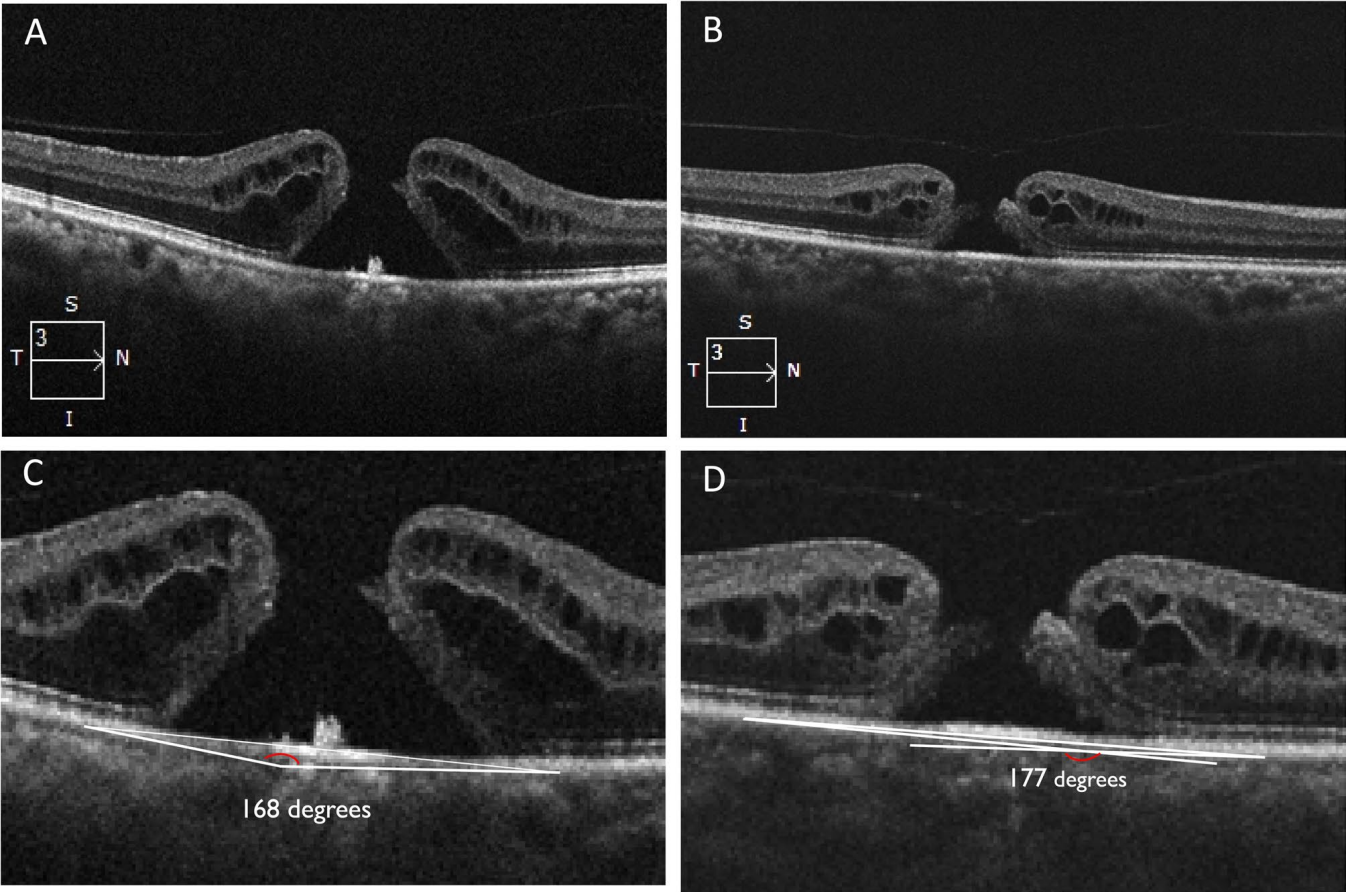
Example showing a greater hole margin tangent apical angle leading to higher basal hole diameter and hole height (**A**, **C**) compared to eyes with flatter hole margin tangent apical angle (**B**, **D**)

There is a great variability in the size and shape of idiopathic macular holes. While size of the MH is important for determining the prognosis in terms of closure and final visual acuity after surgery [16], the shape of the macular hole is more associated with the pattern or orientation of apposition of the hole edges [6]. In a previous study, Terasaki et al. demonstrated fair correlation between the ocular shape index and the horizontal and vertical hole diameter in stage 4 holes, but did not find any significant association with the final visual acuity on multivariate regression [11]. In our study, using a simpler method, we found that there was a negative correlation between the hole margin tangent angle, the tangent angle at 6 mm point, and the absolute BHD and hole height values, both in horizontal and vertical meridian. This meant that a steeper curvature (lesser tangent apical angle) leads to more detachment of the MH margins, leading to larger BHD, akin a MH seen with steep posterior staphyloma (Fig. 5). Surprisingly, we did not find any correlation with the absolute MLD values. We believe there could have been other factors like duration of disease or cystoid changes at the margin of the hole, that may have been responsible of the MLD size (presence of ERM and operculum which could also affect the size, were excluded from the study). This could also explain why in clinical practice, a greater BHD is often seen in macular holes of myopic eyes with staphyloma compared to those in non-myopic eyes.

Apart from the inter-individual variations in posterior pole tangent angle, we realised that the tangent angles varied even between the horizontal and vertical meridian of the same eye. On correlating this ratio of the horizontal to vertical tangent apical angle below the hole margin, with the ratio of MLD, we found a good positive correlation. The vertical tangent apical angles in the central 1 mm were slightly steeper than the horizontal meridian (although not statistically significant). On the other hand, the curvature was much steeper at the 6 mm point in horizontal meridian. This implies that the curvature is flatter near the fovea in the horizontal meridian, but tends to become steeper as we move away from the fovea. The difference in the steepness in both meridians could also explain the wide variability in macular hole shapes in the general population.

The prognostic significance of the macular hole angle has always been doubtful [4, 14]. However, the angle analysed in previous studies has always been the horizontal one. In this study, we analysed each of the nasal, temporal, superior and inferior angles separately. Similar to the previous studies, we did not find any association of the horizontal angles with the type of macular hole closure. However, a higher superior MH angle, along with higher superior to inferior tangent base angle ratio, showed a good association with predilection to a type 1 closure. In other words, a smaller hole angle, with a flatter superior curvature would facilitate a closure of hole margins on the RPE before apposing with the opposite hole margin, resulting in a type 2 closure. The average vertical angle also showed good correlation with the vertical hole height, which could influence the macular hole index values indirectly.

Apart from the retrospective study design, the less number of eye that had a well-documented follow up after surgery, was one of the major limitations. Also, there is always some degree of surgeon-surgeon variation in the surgical technique, which might have had an influence on the closure. Third, the macular holes included in the study was quite large. This could have due to exclusion of holes with operculum, that would have led to exclusion of many small holes. Nonetheless, this is the first study to analyse the effect of posterior pole steepness (tangent angle) on the shape and size of idiopathic macular holes, and is one of the biggest strengths.

## Conclusion

In conclusion, the posterior pole tangent angle appears to have a significant effect on the BHD and vertical hole height. Higher superior hole angle and higher superior tangent angle appears to favour a better apposition of hole edges leading to a type 1 hole closure. These finding provide an insight into the formation of macular holes and the process of macular hole closure, though further validation, with the help of larger prospective studies, will be required.

## Data Availability

The data that support the findings of this study are available from the corresponding author, N.K.S., upon reasonable request.

## Abbreviations

MH: Macular holes
MLD: Minimum linear diameter
HH: Hole height
BHD: Basal hole diameter
OCT: Optical coherence tomography
ERM: Epiretinal membrane
HA: Hole angle
RPE: Retinal pigment epithelium
ILM: Internal limiting membrane
SD: Standard deviation
logMAR: logarithm of minimum angle of resolution
IQR: Interquartile range
ICC: Interclass correlation

## References

[1] Tewari A, Almony A, Shah GK. Macular hole closure with triamcinolone-assisted internal limiting membrane peeling. Retina. 2008;28(9):1276–9. 18626417 10.1097/IAE.0b013e31817d8be1

[2] Sakaguchi H, Ohji M, Oshima Y, Ikuno Y, Gomi F, Maeda N et al. Long-term follow-up after vitrectomy to treat idiopathic full-thickness macular holes: visual acuity and macular complications. Clin Ophthalmol 2012:1281–6. 10.2147/OPTH.S34629 PMC3422155 22927740

[3] Jordan F, Jentsch S, Augsten R, Strobel J, Dawczynski J. Study on the time course of macular pigment density measurement in patients with a macular hole–clinical course and impact of surgery. Klin Monbl Augenheilkd. 2012;229(11):1124–9. 22961042 10.1055/s-0032-1315250

[4] Venkatesh R, Mohan A, Sinha S, Aseem A, Yadav NK. Newer indices for predicting macular hole closure in idiopathic macular holes: A retrospective, comparative study. Indian J Ophthalmol. 2019;67(11):1857–62. 31638049 10.4103/ijo.IJO_364_19 PMC6836585

[5] Unsal E, Cubuk MO, Ciftci F. Preoperative prognostic factors for macular hole surgery: which is better? Oman J Ophthalmol. 2019;12(1):20–4. 30787530 10.4103/ojo.OJO_247_2017 PMC6380157

[6] Sahoo NK, Suresh A, Patil A, Ong J, Kazi E, Tyagi M, et al. Novel En face OCT-Based closure patterns in idiopathic macular holes. Ophthalmol Retina. 2023;7(6):503–8. 36584899 10.1016/j.oret.2022.12.012

[7] Baba T, Tanaka S, Nizawa T, Oshitari T, Yamamoto S. Scleral imbrication combined with Pars plana vitrectomy without internal limiting membrane peeling for myopic schisis. Retina. 2016;36(10):1927–34. 27031526 10.1097/IAE.0000000000001023

[8] Minami S, Ito Y, Ueno S, Kataoka K, Takeuchi J, Ito H, et al. Analysis of macular curvature in normal eyes using swept-source optical coherence tomography. Jpn J Ophthalmol. 2020;64:180–6. 32040660 10.1007/s10384-020-00721-8

[9] Müller PL, Kihara Y, Olvera-Barrios A, Warwick AN, Egan C, Williams KM, et al. Quantification and predictors of OCT-Based macular curvature and Dome-Shaped configuration: results from the UK biobank. Invest Ophthalmol Vis Sci. 2022;63(9):28. 36006653 10.1167/iovs.63.9.28 PMC9428363

[10] Park UC, Ma DJ, Ghim WH, Yu HG. Influence of the foveal curvature on myopic macular complications. Sci Rep. 2019;9(1):16936. 31729424 10.1038/s41598-019-53443-4 PMC6858376

[11] Terasaki H, Yamashita T, Funatsu R, Nomoto S, Fujiwara K, Shiihara H, et al. Effect of the macular shape on hole findings in idiopathic macular hole differs depending on the stage of the macular hole. Sci Rep. 2023;13(1):15367. 37717123 10.1038/s41598-023-42509-z PMC10505151

[12] Gass JDM. Stereoscopic atlas of macular diseases. *Diagnosis and treatment*. 1987:728– 33.

[13] Steel DH, Downey L, Greiner K, Heimann H, Jackson TL, Koshy Z, et al. The design and validation of an optical coherence tomography-based classification system for focal vitreomacular traction. Eye (Lond). 2016;30(2):314–.– 24; quiz 25. 26768921 10.1038/eye.2015.262 PMC4763137

[14] Chhablani J, Khodani M, Hussein A, Bondalapati S, Rao HB, Narayanan R, et al. Role of macular hole angle in macular hole closure. Br J Ophthalmol. 2015;99(12):1634–8. 25995300 10.1136/bjophthalmol-2015-307014

[15] Chen C, Du R, Xie S, Lu H, Xiong J, Wang Y, et al. Longitudinal study of changes in inner scleral curvature patterns and development of posterior staphylomas in highly myopic eyes of children and adolescents. Retina. 2022. 10.1097. 10.1097/IAE.0000000000003681 36727805

[16] Ch’ng SW, Patton N, Ahmed M, Ivanova T, Baumann C, Charles S, et al. The Manchester large macular hole study: is it time to reclassify large macular holes? Am J Ophthalmol. 2018;195:36–42. 30071212 10.1016/j.ajo.2018.07.027

